# Advances in ultrasound for preoperative molecular subtyping of malignant breast tumors

**DOI:** 10.3389/fonc.2026.1774951

**Published:** 2026-04-01

**Authors:** Dao-Rong Hong, Chun-Yan Huang

**Affiliations:** 1 Department of Ultrasonography, The Second Affiliated Hospital of Fujian Medical University, Quanzhou, Fujian, China; 2 Department of General Practice, The Second Affiliated Hospital of Fujian Medical University, Quanzhou, Fujian, China

**Keywords:** breast ultrasound, contrast-enhanced ultrasound, deep learning, elastography, HER2, luminal, molecular subtype, radiomics

## Abstract

Breast cancer molecular subtyping (luminal A, luminal B, HER2-enriched, and triple-negative) guides systemic therapy selection and prognostication, yet is still determined primarily by invasive tissue sampling. Over the last decade, ultrasound has progressed from descriptive B-mode signs to quantitative vascular, mechanical, and computational phenotyping intended to support preoperative subtype inference and biomarker-related risk stratification. This review synthesizes recent advances in conventional ultrasound feature analysis, elastography, contrast-enhanced ultrasound (CEUS), microvascular imaging, radiomics, and deep learning, with emphasis on methodological rigor, interpretability, and clinical translation. Across cohorts, vascularity-related signals (CEUS perfusion metrics and superb microvascular imaging vascular index), stiffness measurements from shear-wave elastography, and multiparametric machine-learning models repeatedly show subtype-discriminative potential. However, heterogeneity in reference standards, single-center retrospective designs, class imbalance, and limited external validation remain major barriers. We outline a roadmap toward clinically deployable ultrasound-based subtyping—prioritizing standardized acquisition, prospective multicenter evaluation, uncertainty-aware and interpretable AI, and multimodal integration with clinicopathologic and genomic context. Importantly, subtype separability should be interpreted as probabilistic discrimination of operational labels rather than biological determinism; mechanistic origins remain largely unknown and require dedicated radiologic–pathologic/radiogenomic validation.

## 1 Introduction

Molecular subtyping provides a pragmatic bridge between breast cancer biology and patient management, informing endocrine therapy, anti-HER2 strategies, chemotherapy, and emerging immunotherapy approaches. Despite its clinical centrality, subtype determination remains dependent on immunohistochemistry (IHC) and/or gene-expression assays, both of which require tissue and may be influenced by spatial heterogeneity and sampling constraints. Imaging-based phenotyping offers an attractive complementary route to characterize tumor biology across the whole lesion and its peritumoral context. Among imaging modalities, ultrasound is widely accessible, repeatable, and well suited for longitudinal monitoring, especially in younger women and dense breasts. Yet its operator dependence and historically qualitative vocabulary have limited its capacity to serve as a robust biological surrogate.

**Figure 1:**
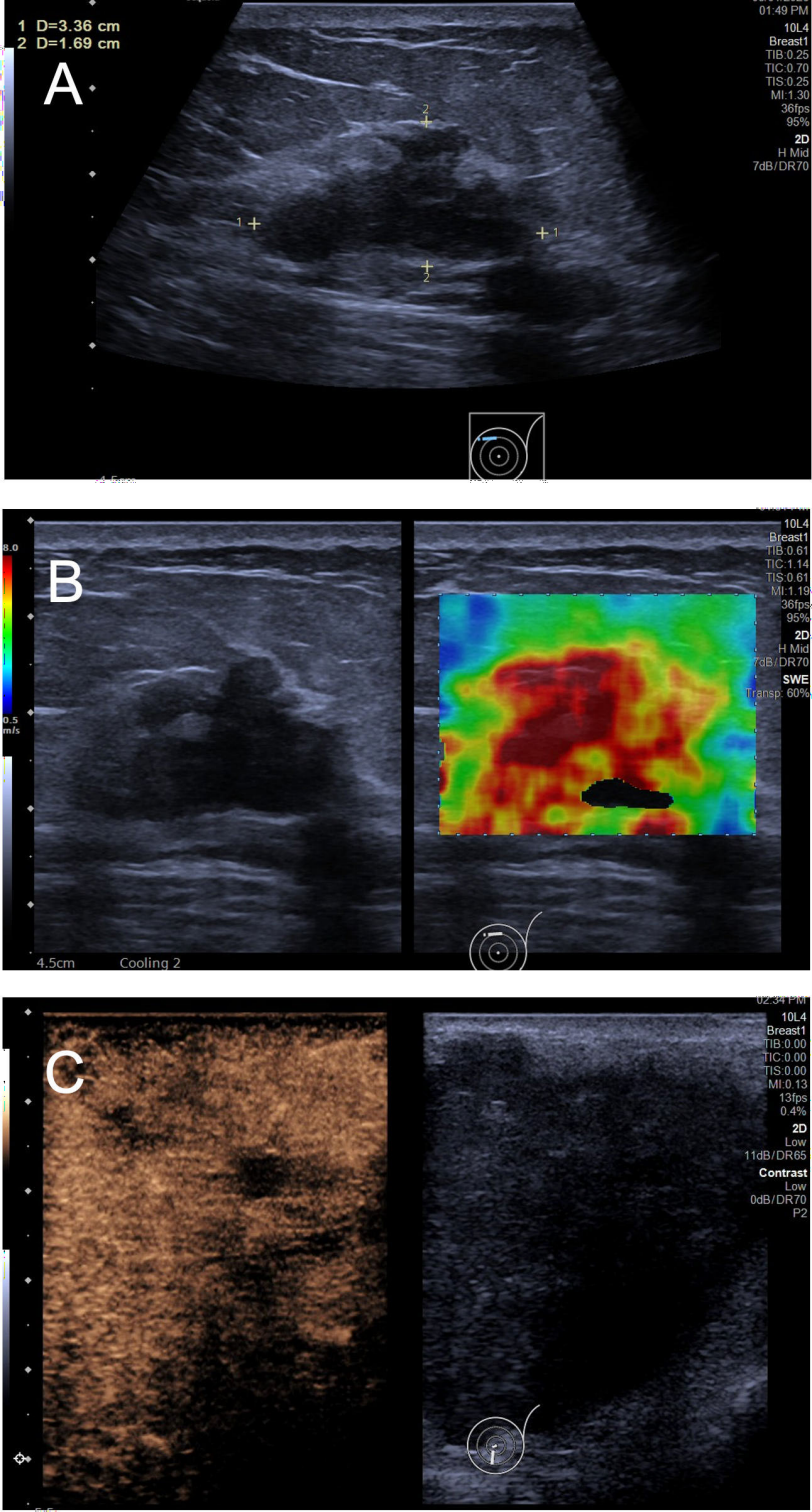
Representative multimodal ultrasound phenotypes reported to correlate with breast cancer molecular subtype labels. **(A)** B-mode ultrasound showing an irregular mass with spiculated margins and posterior acoustic shadowing (attenuation). **(B)** Shear-wave elastography (SWE) stiffness map with quantitative parameters. **(C)** Early-phase CEUS demonstrating rapid wash-in with heterogeneous enhancement and focal perfusion defects (non-enhancing areas), a malignant perfusion phenotype reported to correlate with subtype labels in some cohorts. Images are de-identified and shown for illustrative purposes. Panels are representative examples and may originate from different patients.

Recent work has expanded ultrasound into a multiparametric platform that combines morphology, biomechanics, perfusion, microvascular structure, and high-dimensional texture representations for noninvasive subtype prediction (see **Figure 1**). Subtype ‘prediction’ should be interpreted as probabilistic inference of operational labels, not biological determinism.Throughout this review, we use ‘subtype prediction’ to denote statistical discrimination of clinically assigned subtype labels, not mechanistic inference: current findings are cohort- and labeling-dependent, and they do not establish biological determinism or one-to-one mapping between imaging phenotypes and molecular pathways. However, predictive discrimination of subtype labels should not be conflated with proven biological correspondence; many features may reflect correlated phenotypes (e.g., grade, necrosis, desmoplasia) or technical factors rather than subtype-specific mechanisms.The field has also shifted toward model interpretability—via feature attribution methods and clinically intelligible signatures—to improve trust and facilitate adoption. In this review, we critically appraise the evolving evidence base from handcrafted ultrasound signs to interpretable AI, highlighting consistent signals, unresolved controversies, and the key steps needed for clinical translation.

## 2 Biological and clinical context of molecular subtyping

Operationally, molecular subtypes are commonly approximated using IHC surrogates (ER, PR, HER2, and Ki-67), classifying tumors into luminal A-like, luminal B-like, HER2-enriched-like, and triple-negative groups. These categories capture broad differences in proliferation, angiogenesis, stromal composition, and immune milieu—properties that can plausibly manifest as distinct ultrasound phenotypes through changes in tumor architecture, desmoplasia, and vascular remodeling; however, such associations are probabilistic and do not imply one-to-one biological mapping or validated surrogacy. Clinical subtyping frameworks continue to evolve, and variation in definitions across institutions and studies remains a nontrivial source of noise in model development and cross-study comparison (1, 2).

Operational nature of subtype labels. In most imaging cohorts, “molecular subtypes” are not genomically determined intrinsic subtypes but IHC-defined surrogate (A-like/B-like) labels based on ER/PR/HER2 with additional criteria (often Ki-67 and/or PR), which vary across institutions, guidelines, and time. Ki-67 assessment also exhibits inter-laboratory and inter-observer variability, and different studies use different cut points (e.g., ~14%, 20%, or higher), making subtype assignment heterogeneous and potentially noisy. Accordingly, in this review we treat subtype labels as an operational reference standard rather than biological ground truth, and we frame ultrasound-based “subtyping” as probabilistic inference of these labels under specific cohort and labeling conditions, not as a biological surrogate or replacement.

## 3 Conventional ultrasound phenotypes: what still matters

Conventional B-mode (grayscale) and Doppler ultrasound remain the clinical backbone of breast lesion assessment. While single descriptors rarely provide sufficient discriminative power, several cohorts suggest they provide clinically meaningful priors that can support subtype inference and improve multiparametric models.

In early work integrating mammography with ultrasound, non–triple-negative tumors were more often associated with irregular or spiculated margins, peripheral echogenic halo, posterior acoustic shadowing, and calcifications, whereas triple-negative cancers were emphasized as morphologically variable and capable of mimicking benign masses (3).

Beyond BI-RADS as a reporting framework, recent analyses highlight that certain “benign-appearing” constellations on B-mode can carry subtype implications. For example, triple-negative cancers have been reported to present more frequently as oval or round masses with circumscribed/indistinct margins, posterior acoustic enhancement, internal heterogeneity, and necrotic or cystic components, reflecting high grade and rapid growth kinetics; in contrast, luminal tumors more often show a desmoplastic phenotype with spiculated/angular margins and posterior shadowing (4–6).

HER2-enriched cancers have been associated in some series with a higher prevalence of calcifications and ductal extension patterns that may be apparent even on ultrasound, and with more prominent internal vascular signals on color/power Doppler, although these associations remain inconsistent across cohorts and equipment settings (4–6).

From a day-to-day practice perspective, these conventional cues may help frame pre-test probability and guide targeted tissue sampling (e.g., ensuring viable solid components are sampled in lesions with cystic/necrotic change), but they should not be over-interpreted as deterministic subtype markers.This principle also applies to advanced modalities and AI-derived features discussed below.

Practical bridging points for clinicians include:

- Apparent circumscription with posterior enhancement and internal heterogeneity/necrosis: consider the possibility of aggressive biology (often reported in triple-negative disease) despite benign-like morphology (6).
- Spiculation/architectural distortion with posterior shadowing: more commonly reported in luminal phenotypes with stromal reaction (4–6).
- Calcifications/ductal extension patterns on ultrasound and conspicuous Doppler flow: may raise suspicion for HER2-enriched tumors in some cohorts, but require cautious interpretation (4–6).

Three-dimensional ultrasonography and automated approaches (e.g., ABVS) extend conventional phenotyping by capturing volumetric patterns, such as retraction phenomena and posterior acoustic behaviors that may enrich subtype prediction beyond 2D signs (7).

## 4 Elastography and tumor mechanics as subtype signals

Mechanical phenotyping by elastography leverages the observation that tumor stiffness may be influenced by stromal content, cellular density, and infiltration patterns. Shear-wave elastography (SWE) has been associated with molecular classification and biomarker status across multiple studies, with some cohorts reporting lower stiffness metrics in luminal A-like tumors and higher stiffness in triple-negative disease, and correlations with proliferation markers (e.g., Ki-67) (8, 9).

Beyond diagnosis, SWE has been explored in clinically adjacent tasks where subtype is a key covariate, including axillary lymph node (ALN) assessment and risk stratification, reinforcing the concept that mechanical phenotypes may correlate with clinically relevant tumor characteristics (often correlated with subtype labels) rather than providing mechanistic proof (10–12).

An important practical question is whether elastography-guided sampling can improve subtype assignment from core needle biopsy, potentially mitigating intratumoral heterogeneity by preferentially targeting biologically informative regions (13). These findings support biological plausibility, but stiffness differences may also be confounded by grade, necrosis, tumor size, and acquisition/ROI choices; therefore, they should be interpreted as correlates rather than mechanistic proof of subtype.

## 5 Vascular phenotyping: CEUS and microvascular imaging

Angiogenesis is a hallmark of aggressive breast cancer phenotypes and offers a mechanistic basis for vascular ultrasound biomarkers. CEUS provides dynamic perfusion information through time–intensity curves and qualitative enhancement patterns, while superb microvascular imaging (SMI) and related techniques quantify low-velocity microvascular flow beyond conventional color Doppler. Nevertheless, vascular phenotypes are not subtype-specific and can reflect multiple correlated processes (e.g., angiogenesis, inflammation, hypoxia, and treatment effects), so reported differences primarily indicate statistical association with subtype labels.

CEUS characteristics have been associated with molecular subtype, and quantitative perfusion parameters have been reported to differ across subtypes—including patterns consistent with higher perfusion or faster enhancement in more aggressive phenotypes (14).

SMI-derived vascular index (VI) and vascular distribution patterns have also been reported to vary by subtype, with studies describing relatively low VI in luminal A-like tumors and higher VI with penetrating patterns in triple-negative disease (15).

Notably, multiparametric models combining CEUS and SMI features can achieve strong subtype classification performance in single-center cohorts, and interpretability tools (e.g., SHAP) help identify which vascular descriptors contribute most to subtype separation (16).

## 6 Radiomics and deep learning: from texture to subtype association

Radiomics transforms ultrasound images into high-dimensional feature vectors capturing intensity distributions, texture, and shape, while deep learning learns representations directly from pixel data. These approaches address a central limitation of handcrafted signs: the biology–phenotype relationship is multidimensional and often subtle.

Ultrasound deep learning models have demonstrated promising diagnostic and subtype classification performance, including multicenter retrospective studies and external testing designs (17–19).

Interpretable machine-learning frameworks that integrate clinical variables with imaging features are particularly attractive for translation. In one study, an interpretable model achieved high discriminative performance for subtype prediction and surfaced imaging features with intuitive clinical meaning (20).

Radiomics has also been applied to biomarker prediction (e.g., ER/PR/HER2/Ki-67-related targets), supporting a broader view of ultrasound as a platform for biological inference beyond discrete subtype labels (21, 22).

Importantly, systematic evidence synthesis indicates that while ultrasound radiomics can provide useful predictions, performance varies substantially across studies and does not consistently outperform expert ultrasound assessment, highlighting the need for standardized pipelines and rigorous validation (23).

Hybrid strategies—combining radiomics with deep learning or adding complementary modalities such as photoacoustic imaging—represent an emerging direction that may better capture vascular and oxygenation biology relevant to subtype separation (24–26). A summary of reported diagnostic performance metrics across representative studies is provided in **Table 1**.

**Table 1:** Summary of reported diagnostic performance of ultrasound-based approaches for molecular subtyping (representative studies).

Modality/approach	Representative study (year)	Task/subtype comparison	Design	N	Validation	AUC	Sensitivity/Specificity
Deep learning (US images)	Zhang et al., 2021 (Front Oncol) (17)	Multiclass subtype (Luminal/HER2/TNBC)	Retrospective, single-center	NR	External test (reported)	0.864–0.960	Sens 85.6%–89.7%; Spec 92.9%
Deep learning (CNN, multicenter)	Jiang et al., 2021 (Eur Radiol) (18)	Multiclass subtype (Luminal/HER2/TNBC)	Retrospective, multicenter	1275	External test (reported)	NR	Acc 80.1% (no sens/spec reported)
Interpretable ML (imaging+clinical)	Ma et al., 2022 (Eur Radiol) (20)	Multiclass subtype (Luminal/HER2/TNBC)	Retrospective, single-center	450	Internal validation	0.971	NR
SMI vascular index	Zhang et al., 2022 (Front Oncol) (15)	Subtype association/discrimination	Prospective, single-center	225	No external validation	0.580–0.600	Sens 41.5%–90.3%; Spec NR
SWE/elastography	Evans et al., 2022 (Breast Cancer) (6)	Subtype discrimination (reported)	Prospective, single-center	NR	No external validation	0.690–0.740	NR
CUS+CEUS radiomics	Gong et al., 2023 (Front Oncol) (24)	Multiclass subtype (Luminal/HER2/TNBC)	NR	170	Cross-validation	NR	Acc 70.2% (AUC/sens/spec NR)
TNBC-focused deep learning	Boulenger et al., 2023 (Med Biol Eng Comput) (25)	TNBC vs non-TNBC	Retrospective, single-center	145	Internal validation	0.860	Sens 86.0%; Spec 86.0%
Photoacoustic/US DL+radiomics	Wang et al., 2025 (Breast Cancer Res) (26)	Luminal vs non-luminal	NR	NR	NR	0.717–0.847	NR
CEUS+SMI + machine learning	Chen et al., 2025 (Gland Surg) (16)	Multiclass subtype (Luminal A/B, HER2, TNBC)	Prospective, single-center	135	Independent test cohort	0.846–0.955	NR (test AUC reported ~0.82–0.87 in abstract)

CEUS, contrast-enhanced ultrasound; CUS, conventional ultrasound; SMI, superb microvascular imaging; SWE, shear-wave elastography; TNBC, triple-negative breast cancer; NR, not reported. Metrics are shown as reported in the cited studies (often ranges across endpoints) and are not directly comparable across heterogeneous subtype definitions and validation designs.

To enable an at-a-glance, study-level comparison of the reviewed literature (modality, sample size, and diagnostic performance for subtype prediction), key studies are summarized (see **Table 2**).

**Table 2:** Study-level summary of key ultrasound-based approaches for breast cancer molecular subtype prediction (as reported).

Study	Modality	N	Subtype task	Validation	AUC	Sensitivity	Specificity	Notes
Jiang 2021 (Eur Radiol; PMID 33226454)	Deep learning (multicenter CNN)	1275	Multiclass subtype (Luminal/HER2/TNBC)	Retrospective (stated); Multicenter: Yes; External validation: Yes	NR	NR	NR	Acc 80.1%
Zhang 2021 (Front Oncol; PMID 33747937)	Deep learning (US images)	NR	Multiclass subtype (Luminal/HER2/TNBC)	Retrospective (stated); Multicenter: No; External validation: Yes	0.864–0.960	85.6%–89.7%	92.9%	—
Ma 2022 (Eur Radiol; PMID 34647174)	Interpretable ML	450	Multiclass subtype (Luminal/HER2/TNBC)	Retrospective (stated); Multicenter: No; External validation: No	0.971	NR	NR	—
Evans 2022 (Breast Cancer; PMID 34780035)	SWE/elastography	NR	Subtype discrimination	Prospective (stated); Multicenter: No; External validation: No	0.690–0.740	NR	NR	—
Zhang 2022 (Front Oncol; PMID 35387128)	SMI vascular index	225	Subtype discrimination/association	Prospective (stated); Multicenter: No; External validation: No	0.580–0.600	41.5%–90.3%	NR	—
PMID 35924158 (Front Oncol. 2022)	AI/Radiomics	NR	Molecular subtype (Luminal/HER2/TNBC)	Not stated; Multicenter: No; External validation: Yes	0.686–0.776	NR	NR	—
PMID 36059623 (Front Oncol. 2022)	Conventional	309	Molecular subtype (Luminal/HER2/TNBC)	Retrospective (stated); Multicenter: No; External validation: No	0.744–0.759	NR	NR	—
Boulenger 2023 (Med Biol Eng Comput; PMID 36542320)	TNBC-focused deep learning	145	TNBC vs non-TNBC	Retrospective (stated); Multicenter: No; External validation: No	0.860	86.0%	86.0%	—
Gong 2023 (Front Oncol; PMID 37287927)	CUS+CEUS radiomics	170	Multiclass subtype (Luminal/HER2/TNBC)	Not stated; Multicenter: No; External validation: No	NR	NR	NR	Acc 70.2%
PMID 39705625 (Med Ultrason. 2025)	AI/Radiomics	NR	Molecular subtype (Luminal/HER2/TNBC)	Not stated; Multicenter: No; External validation: No	0.640–0.890	NR	NR	—
Wang 2025 (Breast Cancer Res; PMID 40993716)	Photoacoustic/US DL+radiomics	NR	Luminal vs non-luminal	Not stated; Multicenter: No; External validation: No	0.717–0.847	NR	NR	—
Chen 2025 (Gland Surg; PMID 41142553)	CEUS+SMI + ML	135	Multiclass subtype (Luminal A/B, HER2, TNBC)	Prospective (stated); Multicenter: No; External validation: Yes	0.846–0.955	NR	NR	AUC 0.846–0.955 (internal validation); AUC ~0.82–0.87 (independent test, abstract)

CEUS, contrast-enhanced ultrasound; CUS, conventional ultrasound; SMI, superb microvascular imaging; SWE, shear-wave elastography; TNBC, triple-negative breast cancer; NR, not reported. Metrics are shown as reported in the cited studies (often ranges across endpoints) and are not directly comparable across heterogeneous subtype definitions and validation designs.

## 7 Extending subtype inference to radiogenomics and immune context

Beyond IHC-defined subtypes, a growing body of work explores whether ultrasound phenotypes can infer genomic alterations and immune states. Studies have linked ultrasound characteristics to common driver alterations (e.g., TP53 and PIK3CA) in specific clinical contexts, pointing toward an ultrasound-enabled radiogenomic layer that could complement tissue testing (27, 28).

Immune markers such as PD-1/PD-L1 vary across subtypes and may shape tumor–stroma and vascular interactions; correlative ultrasound studies suggest that imaging phenotypes could potentially stratify immunobiology-relevant subsets, although evidence remains preliminary and cohort dependent (29). At present, these observations remain hypothesis-generating and require prospective validation with matched tissue assays.

## 8 Clinical translation: where subtyping meets decision-making

For ultrasound-based subtyping to influence care, models must be evaluated against clinically actionable endpoints and workflows. Several studies incorporate molecular subtype as a determinant of imaging performance or response prediction, emphasizing that subtype-aware imaging may refine staging and treatment monitoring.

Examples include the observation that subtype can modulate ultrasound performance for ALN evaluation after neoadjuvant chemotherapy (NAC), and that multiparametric ultrasound may support risk modeling for ALN metastasis (30–33).

Longitudinal and treatment-response applications are particularly compelling: CEUS-based approaches and deep learning on serial ultrasound data have been explored for predicting response and pathologic complete response (34–38).

Finally, ultrasound phenotyping can be extended to clinicopathologic correlations in specific populations (e.g., younger women), which may help tailor imaging strategies and model calibration to subgroup biology (9, 39–47).

Workflow feasibility and value-add. In routine preoperative workups, the incremental value of time-intensive modalities should be judged by whether they change a downstream decision rather than by AUC alone. CEUS adds consumables, IV access, monitoring, and post-processing time; thus, its “value add” is typically highest when it can plausibly reduce a more expensive or less accessible test (e.g., problem-solving when MRI is contraindicated/unavailable), refine assessment of treatment response during neoadjuvant therapy, or improve confidence in vascularity-related phenotypes relevant to aggressive biology. Notably, health-economic estimates in other clinical contexts suggest CEUS can be less costly than CT/MRI in per-scan direct costs, supporting targeted rather than universal use when resources are constrained.

Radiomics/AI pipelines can be clinically feasible if the workflow is largely automated (DICOM export → standardized preprocessing → automated/assisted segmentation with QC → locked model inference) and integrated into PACS/RIS; however, manual segmentation and non-standardized preprocessing rapidly erode feasibility and reproducibility. Recent translational guidance emphasizes external validation, transparent reporting, and clinically meaningful utility analyses as prerequisites for adoption.

Pragmatically, we suggest reserving CEUS or complex radiomics for scenarios with a high likelihood of management impact, such as: (i) neoadjuvant therapy monitoring where early response assessment could alter regimen; (ii) cases in which subtype-linked vascular/mechanical phenotypes may affect the intensity of preoperative staging or sampling strategy; and (iii) settings where substituting or triaging advanced imaging could offset added ultrasound time and cost.

## 9 Methodological challenges, controversies, and a roadmap forward

Despite rapid progress, several issues repeatedly limit interpretability and generalizability.

(i) Heterogeneous reference standards. Subtype definitions depend on IHC thresholds and Ki-67 cutoffs, which vary across centers and over time; this creates label noise that can inflate within-site performance while harming portability. To reduce label noise and improve comparability, future studies should explicitly report the IHC scoring protocol and Ki-67 methodology/cut points, and perform sensitivity analyses across plausible Ki-67 thresholds or alternative surrogate definitions.

(ii) Study design limitations. The literature is dominated by retrospective single-center cohorts with modest sample sizes and limited external validation. Class imbalance—particularly for HER2-enriched and triple-negative categories—can bias model training and reported metrics.

(iii) Acquisition and annotation variability. Differences in scanners, presets, lesion segmentation protocols, and radiologist experience can introduce domain shift. Without standardized acquisition and reporting, pooling evidence and deploying models becomes difficult. Practical steps toward standardized acquisition could be implemented in a tiered manner. First, studies should report a minimal “acquisition card” (vendor/model, probe, center frequency, depth, focal zone, gain/dynamic range, harmonic/compounding settings, and for Doppler: PRF, wall filter, color box size/angle). Second, multicenter quality assurance should incorporate tissue-mimicking and flow test objects to quantify cross-scanner variability (e.g., Doppler flow phantoms aligned with IEC standards) and document stability over time. Third, for shear-wave elastography, community adoption of QIBA-style checklists and phantom-based bias testing can provide a concrete, auditable pathway for acceptance and periodic QC, improving comparability across sites. Finally, cross-vendor phantom “round-robin” studies and public reporting of acquisition presets/QA results would accelerate harmonization and enable more reliable pooling of evidence.

(iv) Interpretability versus overfitting. Feature attribution methods (e.g., SHAP) help translate AI outputs into clinically meaningful narratives, but they do not by themselves guarantee causal biological validity. At present, what biological processes give rise to specific texture- or signal-level signatures remains largely unknown; therefore, mechanistic anchoring with matched histopathology (e.g., stromal content, necrosis, microvessel density), molecular assays, and, where feasible, spatial-omics should be prioritized alongside external validation.Mechanistic anchoring—linking imaging signatures to histopathology, microvessel density, stromal composition, and genomic pathways—remains underdeveloped.

Interpretability is not mechanism. Feature importance and *post-hoc* explanation methods (e.g., saliency maps, SHAP) are valuable for understanding model behavior, sensitivity to inputs, and potential failure modes, but they do not establish biological causality. In the absence of direct co-registration between imaging and histopathology, molecular profiling, or microenvironmental measurements (e.g., stromal composition, necrosis fraction, microvessel density, immune infiltrates), statements that ultrasound features “reflect, “ “encode, “ or “represent” tumor biology remain speculative. Future progress will require studies explicitly designed for mechanistic anchoring, including matched imaging–pathology mapping and, where feasible, spatially resolved assays to test whether specific imaging signatures correspond to reproducible tissue-level substrates.

(v) Clinical utility. Many studies optimize AUC for subtype labels, but fewer evaluate how predictions would change management, reduce biopsies, or improve outcomes when integrated into real workflows.

A forward roadmap should prioritize: prospective multicenter trials with preregistered analysis plans; standardized acquisition and radiomics reporting; uncertainty quantification and calibration; external and temporal validation; and interpretable multimodal models that combine ultrasound with clinical and pathological context. As near-term anchors, we recommend leveraging the QIBA Ultrasound Shear Wave Speed Profile (including its QA checklists) to standardize SWE acquisition/QA, and using open RF benchmarks such as the OASBUD dataset to improve reproducibility of signal-level methods and reconstruction-dependent features.

## 10 Conclusions

Ultrasound-based molecular subtyping has evolved from qualitative pattern recognition to multiparametric, data-driven biologically informed prediction and risk stratification. Consistent signals are emerging—especially from vascular (CEUS/SMI) and mechanical (SWE) phenotyping and from integrative AI models—yet translation is constrained by heterogeneity of labels and acquisition, limited multicenter validation, and uncertain clinical utility. With standardized protocols, rigorous evaluation, and interpretable multimodal modeling, ultrasound has realistic potential to become a practical adjunct for preoperative biological stratification and longitudinal monitoring of malignant breast tumors. Establishing biological correspondence will require dedicated radiologic–pathologic/radiogenomic studies rather than performance metrics alone.
